# Fox’s Atlas of Skin Diseases, with Photographic Illustrations

**Published:** 1888-12

**Authors:** 


### FOX'S ATLAS OF SKIN DISEASES, WITH PHOTOGRAPHIC ILLUSTRATIONS. Second edition. Parts 7 and 8; price, $2.00 each. New York: E. B. Treat & Co.

These two parts of this well-known atlas treat of such diseases as porrigo, erysipelas, furunculus, carbunculus, ulcers, etc Chapter third treats of purpura and scorbutus; chapter fourth, of nævus pigmentosus, lentigo, chloasma, clavus, etc. The plates are, of course, very fine.

